# Cyclical DNA Methylation and Histone Changes Are Induced by LPS to Activate COX-2 in Human Intestinal Epithelial Cells

**DOI:** 10.1371/journal.pone.0156671

**Published:** 2016-06-02

**Authors:** Tiziana Angrisano, Raffaela Pero, Mariarita Brancaccio, Lorena Coretti, Ermanno Florio, Antonio Pezone, Viola Calabrò, Geppino Falco, Simona Keller, Francesca Lembo, Vittorio Enrico Avvedimento, Lorenzo Chiariotti

**Affiliations:** 1 Dipartimento di Biologia, Università degli Studi di Napoli “Federico II”, Monte Sant'Angelo via Cintia 21, 80126 Naples, Italy; 2 Dipartimento di Medicina Molecolare e Biotecnologie Mediche, via S. Pansini, 5, 80131 Naples, Italy; 3 Istituto di Endocrinologia ed Oncologia Sperimentale C.N.R., via S. Pansini, 5, 80131 Naples, Italy; 4 Dipartimento di Farmacia, Università degli Studi di Napoli “Federico II”, via D. Montesano 47, 80131 Naples, Italy; Bellvitge Biomedical Research Institute (IDIBELL), SPAIN

## Abstract

Bacterial lipopolysaccharide (LPS) induces release of inflammatory mediators both in immune and epithelial cells. We investigated whether changes of epigenetic marks, including selected histone modification and DNA methylation, may drive or accompany the activation of COX-2 gene in HT-29 human intestinal epithelial cells upon exposure to LPS. Here we describe cyclical histone acetylation (H3), methylation (H3K4, H3K9, H3K27) and DNA methylation changes occurring at COX-2 gene promoter overtime after LPS stimulation. Histone K27 methylation changes are carried out by the H3 demethylase JMJD3 and are essential for COX-2 induction by LPS. The changes of the histone code are associated with cyclical methylation signatures at the promoter and gene body of COX-2 gene.

## Introduction

Inflammation, specifically proinflammatory prostanoids, greatly contribute to shape tumor microenvironment and to neoplastic progression [1]. Bacterial lypopolisaccharide (LPS) induces epithelial and immune cells to release mediators such as prostaglandins, which are synthesized by cyclooxygenase-2 (COX-2). COX-2 is directly involved in the development of chronic inflammation, including inflammatory bowel disease [2, 3]. COX-2 is an immediate-early response gene induced locally by LPS and pro-inflammatory cytokines [4, 5]. COX-2 expression levels are upregulated in a significant fraction of colorectal adenomas and adenocarcinomas [6–8]. COX-2 products induce changes in cell adhesion properties enhancing the metastatic potential. High levels of COX-2 are associated with poor prognosis [9, 10]. Regular use of COX inhibitors greatly reduces the relative risk of developing colon cancer and contrasts cancer stemness [11, 12]. COX-2 expression is regulated by epigenetic mechanisms. For example, COX-2 gene is frequently methylated and silenced in a subset of colorectal tumors (CIMP+ tumors) [13]. Also, in chronic inflammation induced by *Helicobacter pylori* (*Hp*) infection, COX-2 is modulated by DNA methylation [14]. At this stage, mechanisms underlying LPS-induced COX-2 gene modulation and programming have been only partly addressed. However, epigenetic changes in host cells induced by LPS have been recently investigated [15–20]. We have previously shown that *Hp* induces specific epigenetic events at COX-2 and iNOS promoter regions in the first phases of exposure of gastric cells to *Hp* [18, 19]. Furthermore, in intestinal epithelial cells LPS activates IL-8 gene expression by inducing rapid histone modifications in the IL-8 promoter chromatin [17].

In this work, we investigated whether LPS exposure affects epigenetic signatures at COX-2 gene in intestinal epithelial cells. We show that LPS induces cycles of methylation in the DNA and in histone H3 around the COX-2 promoter. Specifically, methylation cycles of H3 lysine 27, a repressive mark associated with transcription inhibition, are induced by LPS and carried out by the H3K27 demethylase JMJD3 to activate COX-2 gene in intestinal epithelial cells.

## Materials and Methods

### Cell culture and treatments

The human intestinal epithelial cell lines HT-29 (Organism *Homo sapiens*, human/tissue colon/disease: colorectal adenocarcinoma) was purchased by ATCC^®^HTB-38^™^ [17] were grown in Dulbecco’s Modified Eagle’s Medium supplemented with 10% fetal bovine serum (Life Technologies), 2 mM glutamine, penicillin (25 U/mL) and streptomycin (25 mg/mL) in a 5% CO_2_ atmosphere at 37°C. Cells were pretreated with Human interferon-γ (IFN-γ) (Roche Applied Science, Germany) 10 ng/ml for 12 hours or control medium as previously described in [21], washed, and then stimulated with LPS 50 ng/ml at different times. LPS (*Escherichia coli*, O55:B5) were purchased from Sigma-Aldrich (St. Louis, MO) and reconstituted in endotoxin-free water.

### Databases

Gene sequences were retrieved by the Ensembl database: COX-2, accession number ENST00000367468.

### Quantitative RT-PCR analysis

Total RNA was isolated with RNeasy extraction kit QIAGEN (Qiagen,GmBh) according to the manufacturer instructions. 1 μg of total RNA of each sample was reverse-transcribed with QuantiTect^®^ Reverse Transcription (Qiagen) using an optimized blend of oligo-dT and random primers according to the manufacturer’s instructions as described in [22]. Quantitative PCR amplifications were performed using QuantiTect SYBR Green (Qiagen) in a Chromo4 Real Time thermocycler (BIORAD). Following primers were used for COX-2 cDNA amplification: (COX2F) 5’-TCACGCATCAGTTTTTCAAGA-3’ and (COX2R) 5’-TCACCGTAAATATGATTTAAGTCCAC-3’; EZH2fw 5’-TGTGGATACTCCTCCAAGGAA-3’ and EZH2rv 5’-GAGGAGCCGTCCTTTTTCA-3’; Primers for evaluation of JMJD3 expression were purchased from Qiagen (Hs_JMJD3_1_SG; Quantitect Primer Assay, QIAGEN); G6PD and 18S rRNA genes were used as housekeeping genes for PCR reaction: G6F (forward) 5’-ACAGAGTGAGCCCTTCTTCAA-3’ and G6R (reverse) 5’-GGAGGCTGCATCATCGTACT-3’ and 18SF: (forward) 5’-GCGCTACACTGACTGGCTC-3’ and 18SR (reverse) 5’- CATCCAATCGGTAGTAGCGAC-3’. The quantitative PCR conditions were: 95°C for 15 min followed by 40 cycles of 95°C for 15 s, 59°C for 30 s, and 72°C for 30 s. Calculations of relative expression levels were performed using the 2^−ΔΔCt^ method [23] and take into account the values of at least three independent experiments.

### Quantitative ChIP analysis

ChIP analysis was performed as previously described [24] using the EpiQuikTM chromatin immunoprecipitation kit from Epigentek Group Inc. (Brooklyn, NY). Specifically, 0.5 × 10^6^ cells treated or untreated were grown to 80%-90% confluency on a 100 mm plate, then trypsinized, collected and washed with PBS. The cells were cross-linked in fresh culture medium with 1% formaldehyde and blocked with 1.25 M Glycine solution. After washing with PBS (2x) the samples were immunoprecipitated with the antibodies according the manufacturers’ instructions. Antibodies used for Protein-DNA immunoprecipitation were: anti-H3-Acetyl (Upstate cat#06–599), anti di-methyl-H3K9 (Upstate cat#07–214), anti tri-methyl-H3K27 (Upstate cat#07–449, Biotechonology, Dundee; UK) anti-di-methyl-H3K4 (Abcam Inc. cat#Ab7766), anti-JMJD3 (ABGENT, cat#AP1022a, San Diego; CA), anti-EZH2 (Cell Signaling TECHNOLGY, cat#AP1022a, Danvers, MA) and normal mouse IgG as a negative control antibody. DNA samples were subjected to quantitative PCR analyses, using SYBR^®^Green Taq (Qiagen) in a Chromo4 Real Time thermocycler (BIORAD). Amplification of the COX-2 promoter was performed using the primers: PCXF 5’-AAGGGGAGAGGAGGGAAAAATTTGTG-3’ (position from nucleotides -89 to -114) and PCXR, 5’-GAGGCGCTGCTGAGGAGTTCCTG-3’ (position from nucleotides +44 to +66). The quantitative PCR conditions were: 95°C for 15 min followed by 40 cycles of 95°C for 15 sec, 62°C for 30 sec, and 72°C for 30 sec. The signal obtained by precipitation with the control IgG was subtracted from the signals obtained with the specific antibodies. Results are expressed as percentage of the input, and calculations take into account the values of at least three independent experiments.

### Gene silencing by RNA interference

HT-29 cell lines were plated at a density of 50% per 6-well plates 24 hrs before transfections. Cells were transfected with JMJD3 siRNA (Qiagen, Hs_jmjd3_2, SI00449827, target sequence: cagcaggaatgccaaggtgaa) or AllStars negative Control siRNA (Qiagen, 1027281) at a final concentration of 5nM using Lipofectamine^®^ Transfection Reagent (Invitrogen, Carlsbad, CA, USA) according to the manufacturer’s instructions. Twenty-four hours after transfection, cells were treated with LPS (50 ng/ml), then harvested at the indicated time points and subjected to mRNA and ChIP analyses as described above.

### DNA methylation analysis by MALDI-TOF

#### Sodium bisulfite treatment

Genomic DNA was isolated with DNeasy extraction kit (Qiagen) according to the manufacturer instructions. Sodium bisulfite conversion was performed using EZ DNA Methylation Kit (Zymo Research, CA USA). One μg for each sample of genomic DNA, eluted in 30 μL of H_2_O, was used for bisulfite treatment. Amplicons used for methylation analysis were obtained from 50 ng of bisulfite treated genomic DNA.

#### MALDI-TOF analysis

We used SEQUENOM MassARRAY platform for DNA methylation analysis. PCR primers to analyze human COX-2 (Upper and Lower strand), designed by using Met*Hp*rimer (www.urogene.org/met*Hp*rimer), were: LCXF, 5’-aggaagagagGGAGTATTGGGATAGATTTAGGAG-3’ and LCXR, 5’-cagtaatacgactcactatagggagaaggctTACCCCCTCAACACCCAAATT-3’, to analyze the region from -218 to +277; UCXF, 5’-aggaagagagTTTTTGTTCCCAAATTGGGGTAGTTTTTTG-3’ and UCXR, 5’-cagtaatacgactcactatagggagaaggctACTAAAATAAACCCAAAAAATCAAAAC-3 to analyze the region from -213 to +272; for reverse primer, an additional T7 promoter tag for *in vitro* transcription was added, as well as a 10-mer tag on the forward primer to adjust for melting-temperature differences. Sequences of these tags are indicated in lower case. The reaction protocol was previously described [25]. Mass spectra were acquired by using a MassARRAY Compact MALDI-TOF (Sequenom) and spectra's methylation ratios were generated by the Epityper software v1.0 (Sequenom).

### Statistical Analysis

Statistical significance between groups was assessed by Student's *t* test. Data are expressed as means Å ± standard deviation (SD). All experiments were repeated at least three times. A *p* value < 0,01 and 0,05 was considered to be statistically significant.

## Results

### LPS induces COX-2 gene in HT-29 intestinal cells

**Fig 1:**
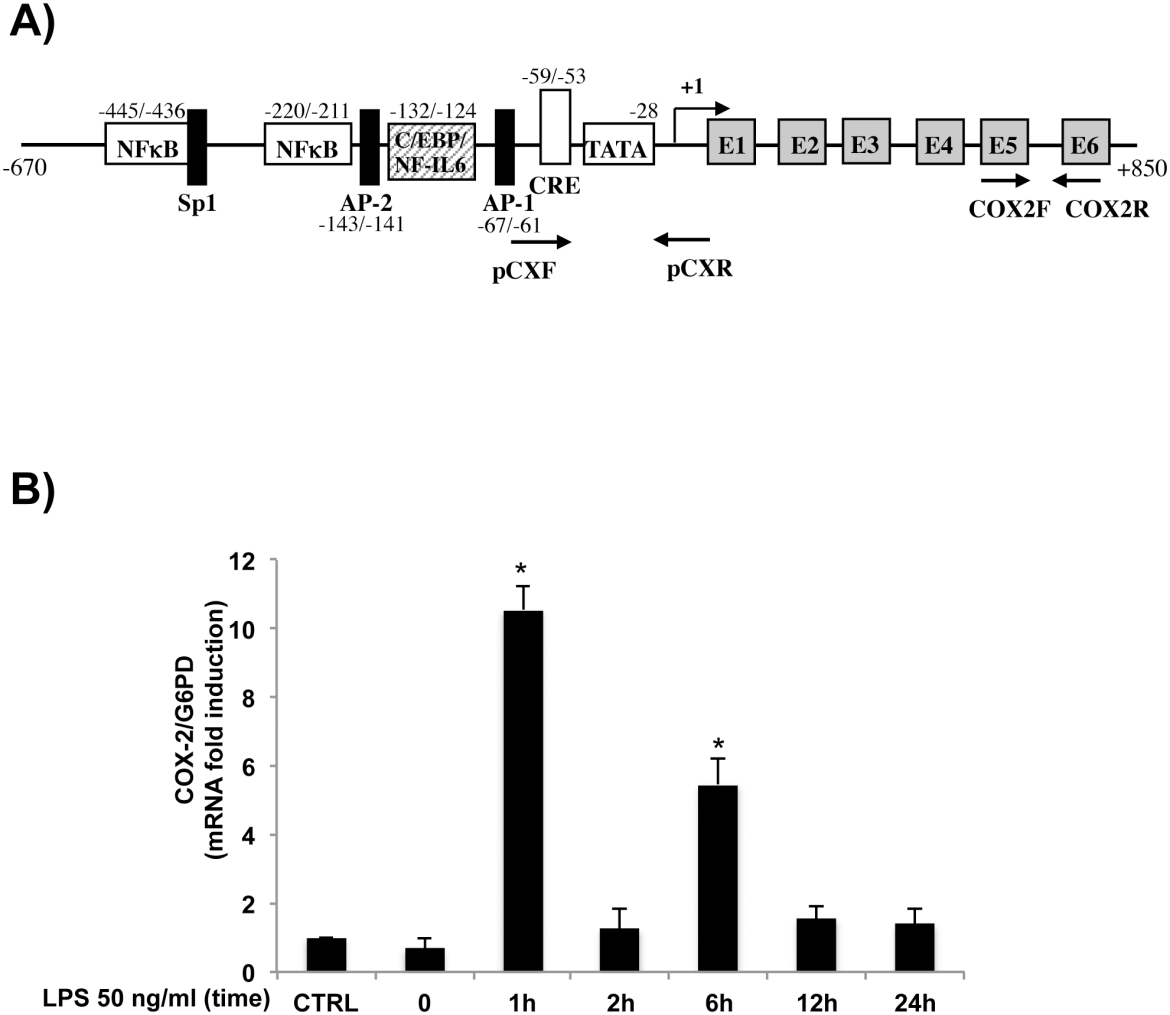
Induction of COX-2 expression in HT-29 cell line by LPS. (A) Schematic representation of human COX-2 gene. Location of primers used for quantitative qRT-PCR (COX2F and COX2R) and ChIP assays (pCXF and pCXR) are indicated. Canonical putative binding sites in the COX-2 promoter region are indicated: NFκB, Sp1, activator protein-2 (AP-2), CCAAT/enhancer-binding protein (C/EBP)/NF-IL6, AP-1, cAMP response element (CRE). TATA box and exon regions (gray boxes) are also indicated. Figure not drawn to scale. (B) Total RNA was isolated at indicated time points after LPS treatment and then subjected to qRT-PCR analysis. Untreated (CTRL) and IFN-γ pretreated (0) HT-29 cells were included as indicated. The COX-2 mRNA levels were normalized to G6PD levels and expressed as relative to untreated control cells (CTRL). Data points represent the average of triplicate determinations ±SD. *, p<0,01.

To investigate COX-2 promoter (Fig 1A) regulation, we first determined the changes in COX-2 mRNA levels induced by LPS in HT-29 human intestinal epithelial cells. HT-29 cells, primed with IFN-γ were treated with LPS, then COX-2 mRNA levels were measured by real-time PCR at different time points (Fig 1B). COX-2 mRNA levels markedly increased in response to LPS, peaking at 1 hour after stimulation with 50 ng/ml LPS (Fig 1B) and then gradually decreased over time. We noticed two peaks of COX-2 mRNA levels at 1h and 6h of LPS treatment, suggesting that LPS induces cyclic transcriptional events to activate COX-2 expression in HT-29 cells.

### LPS-dependent histone modifications at COX-2 promoter

**Fig 2:**
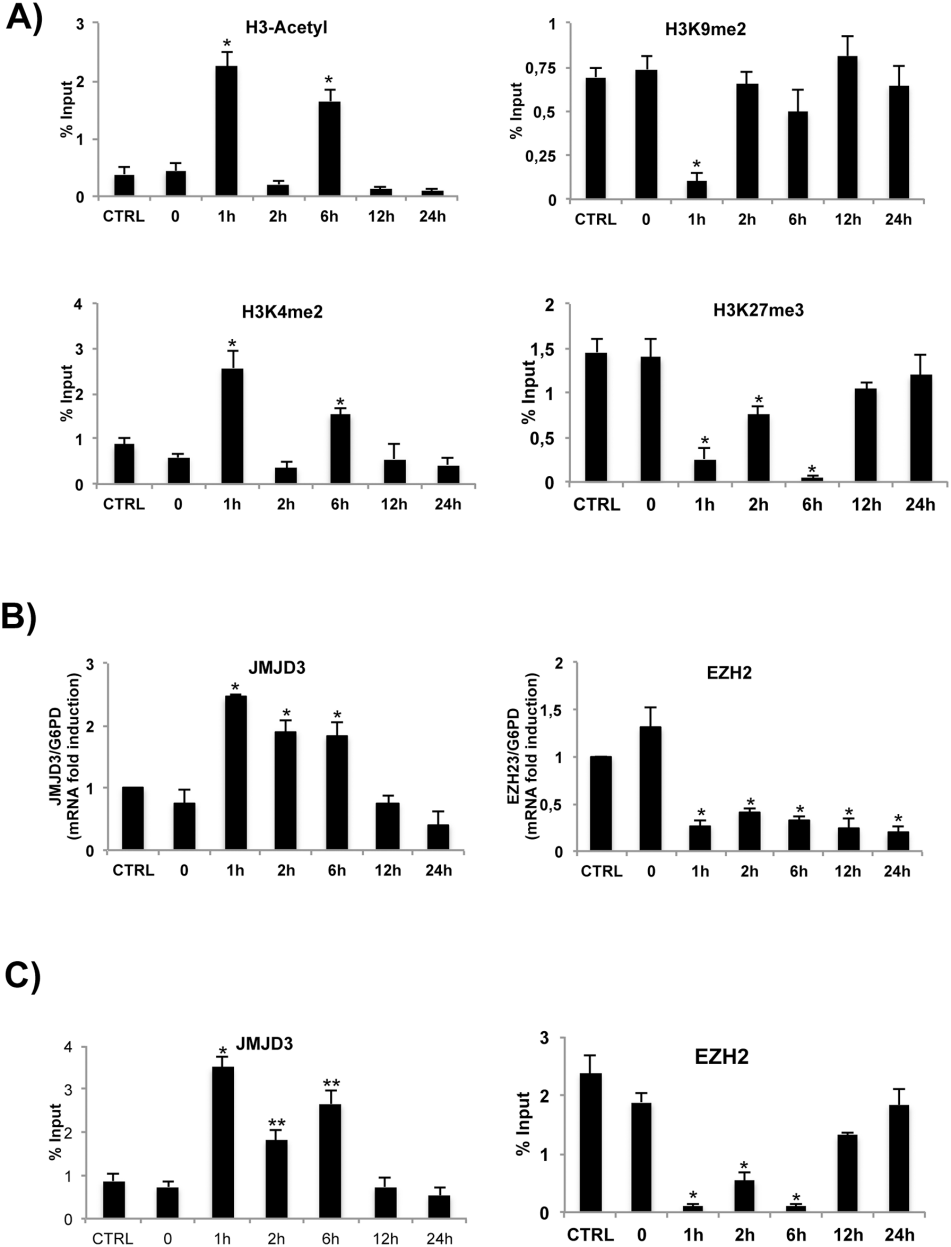
Epigenetic dynamics of LPS-induced COX-2 activation at the COX-2 gene promoter region. (A) LPS induces histone modification changes at the COX-2 gene promoter region. Chromatin changes at COX-2 promoter were analyzed by ChIP assays. Controls include untreated (CTRL) and IFN-γ pretreated (0) HT-29 cells. Chromatin from controls and from HT-29 cells treated with LPS for 1, 2, 6, 12, 24 hours, was immunoprecipitated with the indicated antibodies. Each experiment was repeated at least three times, and the data are presented as percentages of input DNA (mean ± SD). *, p < 0.01; **,p<0,05; (B) Total RNA was extracted at the indicated time points after treatment with LPS and subjected to qRT-PCR analyses of histone demethylases JMJD3 and methyltransferases EZH2. The mRNA levels were normalized to G6PD levels and expressed relative to control cells (CTRL). (C) ChIP experiments were performed with anti-JMJD3 and anti- EZH2 antibodies. COX-2 promoter DNA sequences recovered after the indicated treatments were quantified by real-time PCR. Each experiment was repeated at least three times, and the data are presented as percentages of input DNA (mean ± SD). *, p < 0.01; **,p<0,05.

Histone methylation and acetylation at the COX-2 promoter chromatin following LPS stimulation were analyzed by ChIP assays using anti-acetylated H3, dimethylated H3K4 (H3K4me2), dimethylated H3K9 (H3K9me2) and trimethylated H3K27 (H3K27me3) antibodies (Fig 2A). We found that the chromatin activation marks, H3K4me2 and H3 acetyl, increased significantly at COX-2 promoter chromatin 1 hour after LPS stimulation returning to basal levels at 12–24 hours time points (Fig 2A). Similarly to mRNA accumulation, there are two peaks, at 1 and 6 h after LPS exposure, of H3K4me2 levels (compare Figs 1B and 2A). Conversely, the repressive chromatin marks, H3K9me2 and H3K27me3 displayed an opposite pattern of cyclical changes upon LPS. Specifically, H3K27me3 levels were high in untreated HT-29 cells, decreased at 1 h, partially restored at 2 h and again decreased at 6 h time points after LPS treatment. At 12–24 hours H3K27me3 levels were similar in stimulated and unstimulated cells (Fig 2A). H3K9me2 levels displayed a single cycle, because they decreased early (1h LPS) and returned to the levels of unstimulated cells thereafter. These data suggest that H3K27 methylation changes may be relevant for LPS induction of COX-2 gene and that they may be dependent on changes in the levels or the recruitment of H3K27 methylase and demethylase enzymes (namely, EZH2 and JMJD3) upon LPS stimulation. To define more precisely this point, we analysed first the concentration of these two enzymes following LPS stimulation. Fig 2B shows that EZH2 expression was significantly and rapidly (1 hour) reduced by LPS, while JMJD3 levels were significantly induced by LPS and remained high up to 6 h of LPS stimulation. Second, we analyzed the recruitment of EZH2 and JMJD3 at the COX-2 promoter upon LPS stimulation. Fig 2C shows that JMJD3 recruitment at COX-2 promoter displayed the same cyclical changes of the activation marks (Fig 2A, left panels). As expected, the pattern observed in the recruitment of JMJD3 was reciprocal to that of its natural substrate, H3K27me3 (Fig 2C left panel and Fig 2A right panels). Collectively, LPS induced at COX-2 promoter a cyclical loss and gain of repressive and activation markers, respectively. These oscillatory cycles of epigenetic markers faithfully reflect COX-2 mRNA accumulation in response to LPS.

### JMJD3 depletion inhibits COX-2 induction by LPS

**Fig 3:**
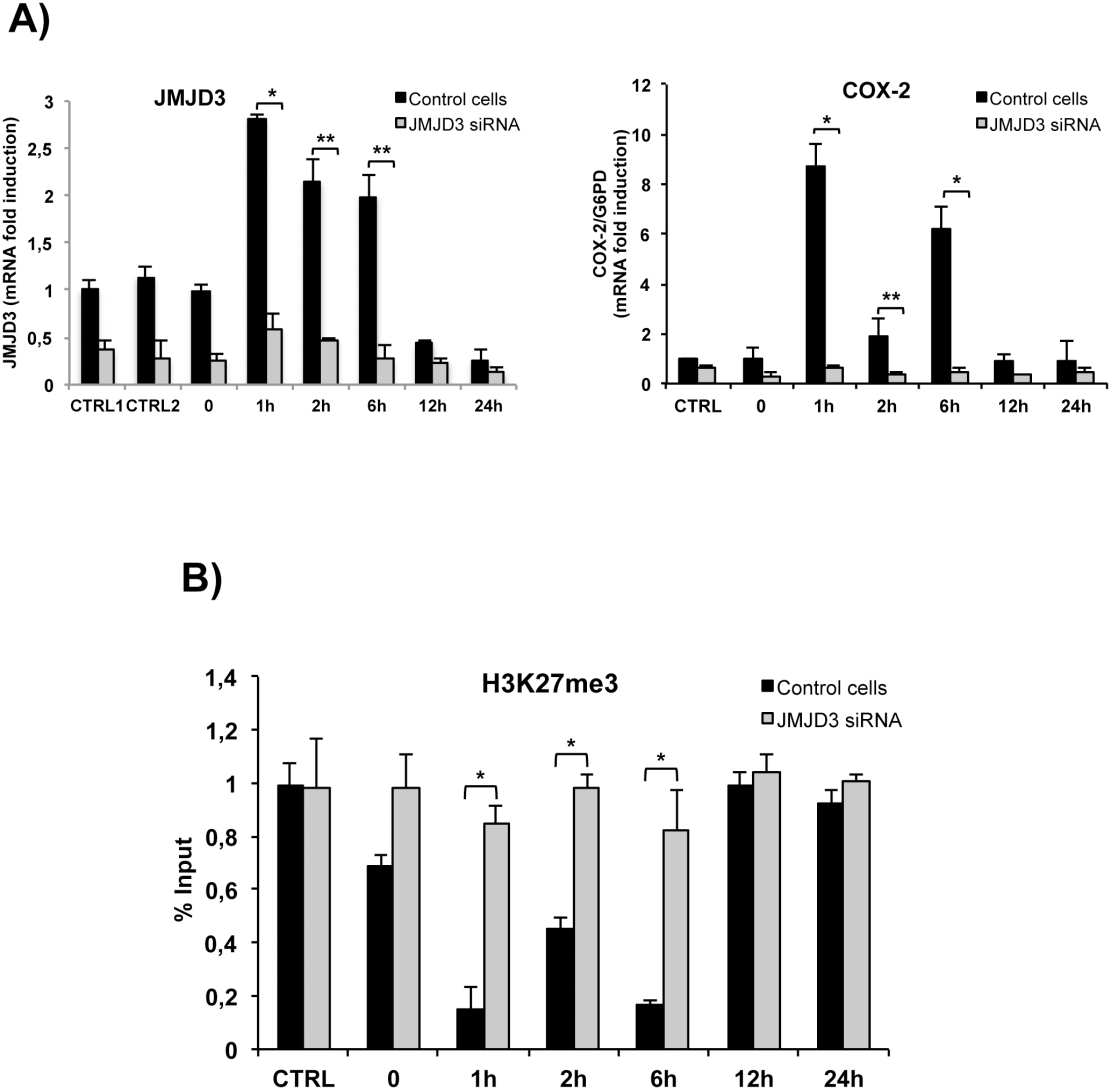
Histone H3K27 demethylase (JMJD3) silencing by siRNA modifies COX-2 expression and histone modifications dynamics during LPS treatment. (A) COX-2 and JMJD3 expression in the presence (grey bars) or absence (black bars) of JMJD3 siRNA during LPS treatment. Time points after LPS administration are indicated. COX-2 and JMJD3 expression was analyzed by qRT-PCR using primers reporeted in Material and Methods section. JMJD3 levels were normalized to G6PD or 18S rRNA levels and expressed relative to control cells (CTRL1 and CTRL2, respectively). COX-2 levels were normalized to G6PD levels and expressed relative to control cells (CTRL). Data points represent the average of triplicate determinations±SD. *p<0.01; **p<0.05. (B) H3K27me3 modulation by LPS treatment at COX-2 promoter was evaluated in the presence of JMJD3 siRNA (grey bars) or negative control siRNA (black bars). Recovered COX-2 promoter DNA sequences after ChIP experiments were quantified by qRT-PCR. Each experiment was repeated at least three times, and the data are presented as percentages of input DNA (mean±SD). *p<0.01. Note that the control experiments were repeated along with siRNA experiments giving results similar to those showed in previous figures.

To investigate the role of JMJD3 demethylase in LPS-induced COX-2 activation, we performed a JMJD3 knock-down experiment. HT-29 cells were transfected with JMJD3 siRNA and JMJD3 and COX-2 mRNA levels were measured by qRT-PCR. In LPS-untreated siRNA-transfected cells, JMJD3 expression decreased more than 70% and did not increase upon LPS treatment (Fig 3). COX-2 induction by LPS was completely inhibited in JMJD3-depleted cells (Fig 3A). ChIP analysis shows that reduction of cellular JMJD3 inhibits the recruitment of the enzyme and the demethylation of H3K27 (Fig 3). Collectively, these data demonstrate that JMJD3 and H3K27 demethylation is required for COX-2 induction by LPS.

### Dynamic changes of CpGs methylation at the COX-2 promoter

**Fig 4:**
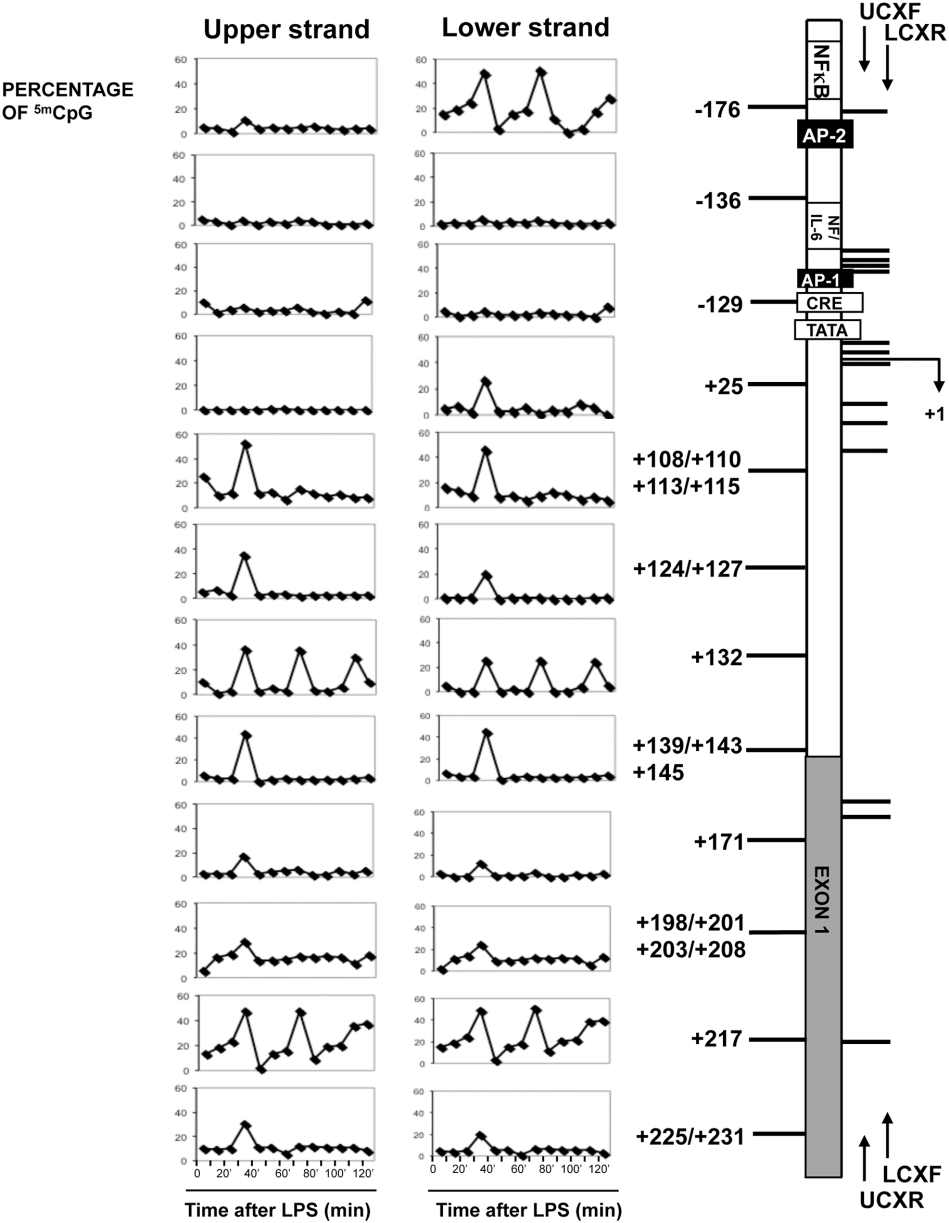
Cycles of DNA methylation in COX-2 gene induced by LPS. Left: percentage of methylation at the indicated CpG sites on upper and lower strands. ‘Time 0’ indicates the methylation status in untreated HT-29 cells. The time scale includes intervals of 10 min for a total of two-hour time course, as indicated. Right: Structure of the human COX-2 gene. Regulatory upstream region (open box), proximal NF-κB binding site, CRE and TATA domains (white boxes), AP-2 and AP-1 (black boxes), exon I (gray box) are indicated. CpG sites (horizontal bars) on the left side were selected because showed significant changes in DNA methylation and the results are shown in the correspondent graphs. TSS (arrow) and the positions of the primers are indicated (UCXF, UCXR for upper strand and LCXF, LCXR for lower strand amplification).

Histone H3 methylation changes can be associated with DNA methylation patterns [26]. Specifically, we have previously described that rapid transient DNA methylation changes at the COX-2 promoter are associated with transcriptional activation and precede histone modifications in gastric cells exposed to *Helicobacter pylori* [18]. Along the same line, here, we investigated the DNA methylation dynamics at COX-2 promoter in LPS-treated HT-29 cells. Twenty-two CpG sites located in the COX-2 promoter region were analyzed by MALDI-TOF MS technique (Sequenom), on both lower and upper strands (Fig 4). DNA methylation was evaluated during the first 2 h of LPS exposure at intervals of 10 minutes in order to be able to detect possible transient changes. Fig 4 shows cycles of methylation/demethylation with a 40 min period upon LPS exposure. CpGs upstream or around the transcription start site (TSS) were methylated and demethylated in a strand specific fashion (-176 and +25 from TSS). Conversely, the CpGs in the body of the gene were methylated-demethylated symmetrically on both strands. It is worth noting that some CpGs were methylated-demethylated only during the first cycle (40 min), while others (+108/115, +124/127, +132, +139/145, +171, +198/208, +217, +225/231) displayed one or more methylation cycles with a 40 min interval between the peaks (Fig 4).

Collectively, these results show very similar oscillatory patterns of DNA and histone methylation changes induced by LPS. The timing of these cycles suggest that DNA methylation changes precede or are synchronous with histone H3K27 demethylation events induced by LPS and carried out by JMJD3. It remains to be seen whether the two events are causally linked.

## Discussion

In the present study, we describe the chromatin and DNA methylation changes occurring at the promoter of COX-2 gene in intestinal epithelial cells as direct consequence of LPS exposure. LPS-dependent COX-2 gene activation was marked by transient cyclic epigenetic events including increase of H3 acetylation and H3K4me2 activation marks, decrease of H3K9me2 and H3K27me3 repressive marks and early cyclic DNA methylation/demethylation events at specific CpG sites of COX-2 gene-regulatory region. Most importantly, we found that JMJD3 demethylase was enriched at COX-2 promoter in concomitance with depletion of H3K27me3 levels and displacement of EZH2, a H3K27 methylase. Finally, the essential role of JMJD3 was assessed by siRNA experiments.

### Cycles of histone H3K27 methylation induced by LPS at the COX-2 promoter

Ours and others’ recent studies demonstrate that bacteria, or their components such as LPS, are able to induce changes in histone modifications in exposed cells, thereby altering the host’s transcriptional program and possibly affecting the host innate immune response [15–20]. However, the majority of the previous studies were carried out in immune cells (neutrophils, monocytes, macrophages and dendritic cells) [27] while it is not well known the response to LPS of epithelial cells, which represent the first barrier against microbes. Moreover, most of studies addressing LPS-induced epigenetic modifications at COX-2 gene were focused on histone phosphorylation and acetylation dynamics and few on histone and DNA methylation [15, 16, 28, 29]. In an early study [30] it was reported that LPS induced IL-12 production by a rapid and specific nucleosome re-organization at IL-12 promoter region in murine macrophages. Transient changes in H3 acetylation and H3K4, H3K9 and H3K27 methylation in IL-8 gene promoter were induced by LPS and pretreatment of HT-29 colon cancer cells with deacetylase inhibitors amplified LPS-induction of IL-8 [17]. A more precise analysis overtime of histone H3K27 methylation at the COX-2 promoter reveals at least 2 cycles of methylation involving H3K27. JMJD3 demethylase seems an important mediator of LPS induced H3-K27 methylation cycles, because depletion of this enzyme severely impairs LPS induction of COX-2 and abolishes H3K27 methylation cycles (Fig 3). However, recruitment of the demethylase (JMJD3) and the loss of the methyltransferase (EZH2) at the COX-2 promoter is steady and progressive (Fig 2B), suggesting that not the concentration but the activation of JMJD3 is cyclical. We propose that methylation cycles of H3K4 and H3K27 follow reciprocal patterns (Fig 2A). Methylation of H3K4 transiently halts demethylation of H3K27 and this allows the ordered recruitment of transcription initiation factors. These events impact also on RNA accumulation, because we observed a cycle also at COX-2 RNA levels following LPS challenge. We wish to stress that we could detect these cycles (RNA included) because the cells have been synchronized in two steps and are responsive to LPS stimulus. With the time 6–12 hours after the initial LPS challenge, transcription stochastically de-synchronizes and the histone methylation-demethylation cycles are not detectable (Fig 2). Finally, the simultaneous presence of H3K9 and H3K27 methylation marks at COX-2 gene promoter region has been previously suggested to contribute to the maintenance of constitutive heterochromatin and more stable gene silencing [31]. However, our data show that LPS stimulation is able to induce rapid and simultaneous loss of both repressive marks at COX-2 promoter.

### Cycles of CpG methylation induced by LPS at the COX-2 gene

The most striking finding presented here is the temporal association between histone H3K27 and H3K4 methylation cycles with methylation cycles of selected CpGs in COX-2 gene. There are similar examples of cyclical DNA methylation in genes induced by estrogens [32, 33] or cyclical histone H3 K9 and K4 methylation in genes induced by retinoic acid [34]. This is the first example of temporal correlation between histone and DNA methylation. Although we have not clarified the mechanistic link between the two methylation events, we note some specific signatures of the CpGs undergoing to methylation cycles around the TSS of COX-2 gene upon LPS challenge. The CpGs in the promoter region (-176 and +25) were transiently methylated in the *minus* strand only, while the CpGs in the body of the gene (+108 to +225) underwent periodic methylation on both strands (Fig 4), similarly to CpGs in estrogen responsive genes [32, 33]. Moreover, some CpGs displayed a single methylation cycle at 40 min of LPS exposure, while others were periodically methylated in two methylation-transcription cycles, or in three cycles (+135 and + 217), with a time interval period of 40 minutes between each cycle (Fig 4). We hypothesize that these epigenetic changes define a specific transcription territory in or around the gene and its regulatory regions. For example, these transient and differential methylation marks may differentially label the regulatory regions and the gene body.

Overall, we believe that the analysis of the epigenetic changes induced by LPS to activate COX-2 provides a specific temporal window on the early phases of transcription. Moreover, because aberrant methylation of COX-2 is frequent in colon cancer [13], it will be very important to investigate whether transient cyclical methylation occurring during transcription cycles may become inaccurate and distorted thus potentially contributing to aberrant methylation changes at COX-2 gene promoter during neoplastic progression.
